# Antrochoanal Polyps: How Long Should Follow-Up Be after Surgery?

**DOI:** 10.1155/2015/297417

**Published:** 2015-08-03

**Authors:** Saisawat Chaiyasate, Kannika Roongrotwattanasiri, Jayanton Patumanond, Supranee Fooanant

**Affiliations:** ^1^Department of Otolaryngology, Faculty of Medicine, Chiang Mai University, Chiang Mai 50000, Thailand; ^2^Clinical Research Center, Faculty of Medicine, Thammasat University, Pathum Thani 12120, Thailand

## Abstract

*Objective*. To investigate the length of follow-up needed to detect recurrence of antrochoanal polyps. *Methods*. A retrospective investigation was performed on patients who had been operated on with a preoperative diagnosis of antrochoanal polyps in Chiang Mai University hospital from 2006 to 2012. *Results and Discussion*. Of the 38 cases of choanal polyps, 27 were adults (71%). The median age was 23.5, ranging from 7 to 64 years old. Eighteen patients were male (47.4%). The origin of choanal polyps was the maxillary antrum in 32 patients. The most common symptom was nasal obstruction (97.4%). The surgical procedures were polypectomy in one child and combined endoscopic and transcanine fossa approach in two adults. The remainder of the patients underwent endoscopic removal of the polyps. The follow-up time ranged from 1 day to 8 years. There were 5 cases of recurrence of which four were in children. The time for recurrence was 1.2 ± 0.6 years (95% CI 0.51, 1.97). *Conclusion*. Antrochoanal polyps are more common in younger patients. Recurrence was significantly higher in children. Follow-up of patients should be for at least 2 years postoperatively in order to detect 95% of recurrence.

## 1. Introduction

The condition of antrochoanal polyps (Killian polyps) is a distinctive clinical disease. It is characterized by polyps originating from the maxillary antrum, which then extend through the natural or accessory ostium into the nasal cavity, choana, and nasopharynx. The maxillary portion is cystic though there are some reports of solid forms (polyps), while nasal and choanal portions are usually solid [1]. Choanal polyps may come from the sphenoid sinus, the nasal septum, and other parts of the nasal cavity [2–4]. Antrochoanal polyps occur as 4–6% of adult polyps [5] and 33% of childhood polyps [6]. The most common presenting symptom is nasal obstruction, either unilateral or bilateral. Other complaints are rhinorrhea, sinusitis, snoring, dysphagia, and so forth. Complete surgical removal of the nasal and antral portion of the polyp is the standard treatment to prevent recurrence. However, in some patients with a small maxillary sinus or in revision cases, the origin of the polyp could not be well identified. This study is to investigate the length of follow-up needed to detect recurrence of polyps in patients. The authors also would like to discern if there are differences in recurrence between children and adults.

## 2. Materials and Methods

A retrospective investigation was carried out on patients who had been operated on with a preoperative diagnosis of antrochoanal polyps in Chiang Mai University hospital from 2006 to 2012. After excluding 6 cases of inverted papilloma and 1 case of maxillary mucopyocele, 38 cases of patients with choanal polyps were included in this study. Clinical data and operative findings were reviewed, and the latest follow-up data were collected. Delayed diagnosis was defined as treating patients with another diagnosis such as sinusitis or allergic rhinitis for more than 3 visits to the outpatient document without recording incidence of polyps. The treatment of antrochoanal polyps was complete surgical removal with either an endoscopic approach alone or an endoscopic approach combined with the transcanine fossa approach.

The data were analyzed using the STATA program version 11.0 (STATA Corporation, Texas, USA). The exact probability test was used for the proportion of the investigative variables between the age groups, and survival analysis was used for evaluating the potential factors affecting recurrence.

The Research Ethics Committee of the Faculty of Medicine of Chiang Mai University approved the study protocol.

## 3. Results

Of the 38 cases of choanal polyps, 27 were adults (71%). The median age was 23.5, ranging from 7 to 64 years. Eighteen patients were male (47.4%). There was no statistical difference in the sex of the age groups (Table 1). The origin of choanal polyps was the maxillary antrum in 32 patients. The other polyps originated from the superior turbinate or sphenoethmoidal recess, totaling 6 adult patients. The most common symptom was nasal obstruction (97.4%), either unilateral (57.9%) or bilateral (39.5%). Positional changing of the obstruction in the supine or lateral decubitus was found in 9 patients (23.7%). Other symptoms were purulent rhinorrhea (71%), pain (26.3%), epistaxis or bloody nasal discharge (13.2%), and sore throat (7.9%). One adult patient who presented with a sore throat and a mass in the oropharynx for 3 days had no nasal obstruction at all. The duration of symptoms ranged from 3 days to 4.5 years, with a median time of 1 year.

When comparing between age groups, the symptoms showed no significant difference. However, purulent rhinorrhea was more common in children (88.8% compared to 66.7%) and pain was more common in adults (29.6% compared to 18.2%). Delayed diagnosis was more common in adults (29.6%) than in children (9.1%).

**Figure 1:**
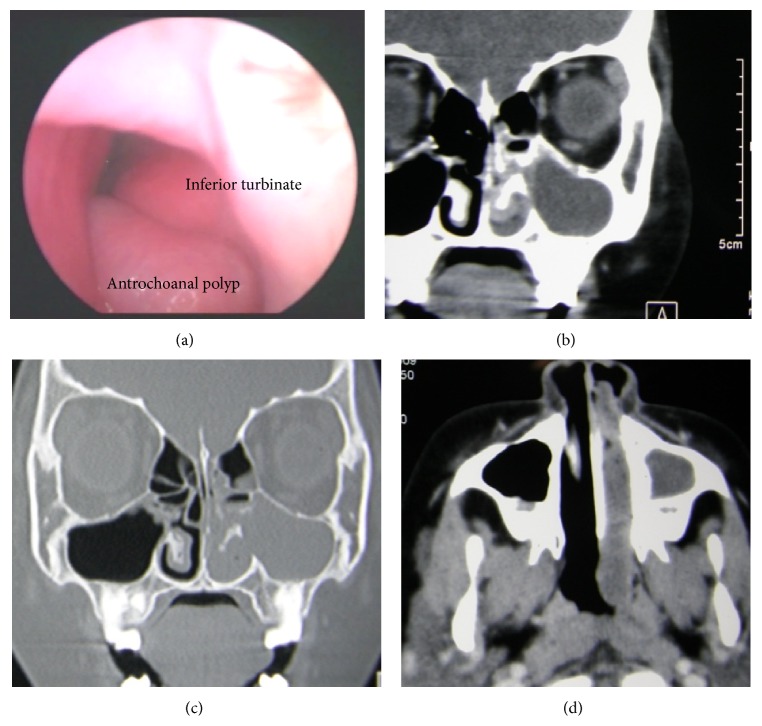
(a) Left nasal cavity endoscopic view showing antrochoanal polyp at inferior meatus CT scans. (b) Coronal view, soft tissue window showing cystic component in the maxillary antrum. (c) Coronal view, bone window showing defect of medial maxillary wall below the inferior turbinate. (d) Axial view, soft tissue window; polyp extending into the nasopharynx.

The surgical procedures were polypectomy in one child and combined endoscopic and transcanine fossa approach in two adults. The remainder of the patients underwent endoscopic removal of the polyps by a middle meatus antrostomy with an operative note of incomplete removal of the maxillary part in the case of one child. All but one polyp extended into the nasal cavity through the middle meatus, via either natural or accessory ostium. Only one case differed in the children, where the polyp extended through the inferior meatus (Figure 1).

The follow-up time ranged from 1 day to 8 years, the median being 1.2 years. Two patients failed to keep appointments for the postoperative care that was scheduled. There were 5 cases of recurrence of which four were in children. The time for recurrence was 1.2 ± 0.6 years (95% CI 0.51, 1.97). The case of recurrence in an adult was where the surgery had been carried out in another hospital. After the recurrence a revision was carried out at CMU and no further recurrence occurred in the one-year follow-up period. The origin of the polyps from all recurrent cases was from the maxillary sinus, while none of sphenoethmoidal polyps recurred.

The sex, age group, and infection were tested as risk parameters for recurrence. The origin of polyps and type of surgical procedure were not tested because of the limited numbers of patients in each subgroup (Table 2). The polyps recurred significantly more in the group of children when compared to that of the adults (*p* = 0.036).

## 4. Discussion

Antrochoanal polyps have been known about for some time; for example, in 1691 a polyp from the antrum of Highmore was mentioned by Fredrik Ruysch. Antrochoanal polyps (ACP) are also known as Killian polyps after Gustav Killian, the doctor who stressed this special type of polyp from the maxillary antrum to choana in 1906 [1]. Though the pathogenesis is still unknown, Berg et al. studied the macro- and microarchitecture of ACPs and suggested that they develop from an expanding intramural cyst, protruding through the maxillary ostium into the nasal cavity [7]. Frosini et al., in the largest study of 200 cases, suggested that they occurred from the combination of an antral cyst and a maxillary ostium obstruction with an association with an anatomical abnormality such as a deviated nasal septum and a turbinate alteration [1]. Mostafa et al., on the other hand, studied 25 cases of ACPs in which only 5% of the antral parts were cystic [8]. They compared the transitional zone of an ACP with chronic sinusitis with nasal polyps and found a higher density of lymphatic markers on the ACP group (88% versus 16%). Mostaf et al. suggested that the lymphatic obstruction might be a process associated with ACP development.

Antrochoanal polyps were found in patients with a wide age range from 5 to 81 years [1, 3, 9]. The patients in this study ranged from 7 to 64 years of age. The median age of 21 years showed that this type of polyp was more common in the younger age group as was found in many previous studies [1, 10–12]. Other studies found that the ACP occurred more commonly in males, but this was not found to be the case in this study. When comparing the clinical presentations and the outcome of treatment in children and adults, there was no statistical significant difference in clinical presentations though infection was more common in the younger age group. The key of successful treatment is complete removal of the polyp from the maxillary origin. The inflammatory mucosa of sinusitis was mentioned as a possible risk of recurrence in some studies as it led to difficulty in identifying the origin in the antrum [9, 13]. The polyps originating from the lateral, anterior, and inferior walls of the maxillary antrum were difficult to view and remove with the transnasal endoscopic approach alone [9–11]. Special instruments or a combined transcanine fossa approach may be needed to complete surgical removal [9, 11, 14–16]. In children, the anatomically narrow sinuses, the nonerupted teeth, and concern of maxillary growth may effect surgeons' decision on the surgical approach, leading to recurrence.

The choanal polyps which originated from the superior turbinate or the sphenoethmoidal recess showed no recurrence. This type of polyp might be different from those developing from the maxillary antrum and is easier to locate and remove from its origin. No other types of choanal polyps in children were found, though several have been reported in other studies [17, 18].

This study found that the age group alone was significantly associated with recurrence (*p* value = 0.036). In other studies, the occasion of recurrence was found as early as 6 months in the cases of incomplete removal [10] to as long as 3 years [3]. Ten percent of our patients did not come back for postoperative evaluation as they lived very far away or came from neighboring countries. In this group of patients, the postoperative cleaning was carried out before discharge to ensure sinus drainage was adequate. The median follow-up time was 1.2 years, though the longest was up to 8 years. The overall time of recurrence in this study was 1.2 ± 0.6 years (95% CI 0.51, 1.97). We suggested monitoring ACP patients for at least 2 years in order to detect 95% of recurrence.

## 5. Conclusions

Antrochoanal polyps are more common in younger patients. Recurrence was significantly higher in children. Follow-up of patients should be for at least 2 years postoperatively in order to detect 95% of recurrence.

## Figures and Tables

**Table 1:** Patient characteristics according to age group.

	Total (38 patients)	Age <15 years (11 patients)	Age ≥15 years (27 patients)	*p* value
Age (year)	Median 23.5			
Mean ± SD	28.1 ± 16.3	10.7 ± 2.6	35 ± 14	
Range	7–64	7–14	15–64	
Sex				
Male : female		6 : 5	12 : 15	0.724
Symptoms				
Nasal obstruction	37 (97.4%)	11 (100%)	26 (96.3%)	1.000
Unilateral		7 (63.6%)	15 (55.6%)	
Bilateral		4 (36.4%)	11 (40.7%)	
None		0	1 (3.7%)	
Positional change	9 (23.7%)	3 (27.3%)	6 (22.2%)	1.000
Progression		10 (90.9%)	17 (63%)	0.124
Purulent rhinorrhea	27 (71.1%)	9 (81.8%)	18 (66.7%)	0.452
Epistaxis	5 (13.2%)	2 (18.2%)	3 (11.1%)	0.615
Pain	10 (26.3%)	2 (18.2%)	8 (29.6%)	0.690
Sore throat	3 (7.9%)	—	3 (11.1%)	0.542
Delayed diagnosis	9 (23.7%)	1 (9.1%)	8 (29.6%)	0.237
Follow-up				
Less than a month	7 (18.4%)	2 (18.2%)	5 (18.5%)	
Median (year)	1.22	1.24	1.20	0.721
Range	1 day–8 years	1 day–7 years	1 day–8 years	
Operation				
Endoscopic polypectomy		1	—	
Endoscopic removal		10	25	
Combined endoscopic and transcanine fossa		—	2	
Origin				
Maxillary sinus		11	21	
Sphenoethmoidal recess/superior turbinate		—	6	
Recurrence		4 (36.4%)	1 (3.7%)	0.019

**Table 2:** Multivariable odds ratios (OR) and 95% confidence interval (CI) of potential factors affecting recurrence of antrochoanal polyps.

Parameters	OR	95% CI		*p* value
Young age group	10.52	1.17	94.71	0.036
Sex	1.03	0.16	6.51	0.978
Infection	1.78	0.19	17.14	0.617
